# Inhibition of *Streptococcus pneumoniae* adherence to human epithelial cells *in vitro* by the probiotic *Lactobacillus rhamnosus* GG

**DOI:** 10.1186/1756-0500-6-135

**Published:** 2013-04-05

**Authors:** Sook-San Wong, Zheng Quan Toh, Eileen M Dunne, E Kim Mulholland, Mimi LK Tang, Roy M Robins-Browne, Paul V Licciardi, Catherine Satzke

**Affiliations:** 1 Pneumococcal Research, Murdoch Childrens Research Institute, Royal Children’s Hospital, Parkville, VIC, Australia; 2 Allergy and Immune Disorders, Murdoch Childrens Research Institute, Royal Children’s Hospital, Parkville, VIC, Australia; 3 Infectious Diseases and Microbiology, Murdoch Childrens Research Institute, Royal Children’s Hospital, Parkville, VIC, Australia; 4 London School of Hygiene and Tropical Medicine, London, UK; 5 Menzies School of Health Research, Charles Darwin University, Darwin, NT, Australia; 6 Allergy and Immunology, Royal Children’s Hospital, Parkville, VIC, Australia; 7 Department of Paediatrics, The University of Melbourne, Parkville, VIC, Australia; 8 Department of Microbiology and Immunology, The University of Melbourne, Parkville, VIC, Australia; 9 Present address: St. Jude Children’s Research Hospital, Memphis, TN, USA

**Keywords:** Probiotic, LGG, Pneumococci, Colonization, *in vitro* model

## Abstract

**Background:**

Colonization of the nasopharynx by *Streptococcus pneumoniae* is considered a prerequisite for pneumococcal infections such as pneumonia and otitis media. Probiotic bacteria can influence disease outcomes through various mechanisms, including inhibition of pathogen colonization. Here, we examine the effect of the probiotic *Lactobacillus rhamnosus* GG (LGG) on *S. pneumoniae* colonization of human epithelial cells using an *in vitro* model. We investigated the effects of LGG administered before, at the same time as, or after the addition of *S. pneumoniae* on the adherence of four pneumococcal isolates.

**Results:**

LGG significantly inhibited the adherence of all the pneumococcal isolates tested. The magnitude of inhibition varied with LGG dose, time of administration, and the pneumococcal isolate used. Inhibition was most effective when a higher dose of LGG was administered prior to establishment of pneumococcal colonization. Mechanistic studies showed that LGG binds to epithelial cells but does not affect pneumococcal growth or viability. Administration of LGG did not lead to any significant changes in host cytokine responses.

**Conclusions:**

These findings demonstrate that LGG can inhibit pneumococcal colonization of human epithelial cells *in vitro* and suggest that probiotics could be used clinically to prevent the establishment of pneumococcal carriage.

## Background

*Streptococcus pneumoniae* (the pneumococcus) is a Gram-positive bacterium that causes serious diseases such as pneumonia, meningitis and acute otitis media. Pneumococcal diseases are a leading cause of childhood morbidity and mortality worldwide, affecting more than three million children under the age of five, and causing an estimated 826,000 deaths in this age group each year [1]. The disease burden is especially high in developing countries [1]. Pneumococcal colonization of the nasopharynx is often asymptomatic, occurs early in life, and is considered a prerequisite for development of pneumococcal disease [2]. In high-risk populations, pneumococci can colonize the nasopharynx within the first few weeks of life [3].

Pneumococcal conjugate vaccines (PCV) provide protection against the serotypes most prevalent in pediatric invasive disease [4]. However, developing countries have a substantial burden of invasive disease from non-vaccine serotypes, and serotype replacement is likely to be more important in these settings [5]. In addition, access to these vaccines is limited in resource-poor countries and colonization often occurs before the first dose of PCV, typically given at two months of age. Early life strategies to reduce or prevent colonization and carriage are urgently needed, particularly in populations with high rates of pneumococcal disease.

Probiotics are defined as “live microorganisms which when administered in adequate amounts confer a health benefit on the host” [6]. They can influence host microbiota and play a role in disease prevention [7]. Probiotics such as *Lactobacillus* and *Bifidobacterium* species are widely used in food products or as food supplements and have been extensively studied in the gastrointestinal tract [8]. Probiotics are believed to benefit the host through several mechanisms including i) inhibition of colonization by pathogenic microorganisms [9], ii) modulation of host immune responses [10] and iii) improvement of epithelial cell barrier integrity [11].

Although less is known about the effects of probiotics in the respiratory tract, evidence that they could be used to prevent disease in this context is mounting [12,13]. For example, lactobacilli have been shown to protect against pneumococcal infection in mice [14-16], and inhibit the invasion of group A streptococci *in vitro*[17]. In humans, administration of a probiotic drink containing *Lactobacillus rhamnosus* GG (LGG), *Bifidobacterium lactis* sp B420, *Lactobacillus acidophilus* 145, and *Streptococcus thermophilus* reduced nasal colonization by Gram-positive pathogens in adults [18]. These studies suggest that some probiotic species can reduce pneumococcal colonization, potentially serving as a safe and cost-effective complementary strategy to immunization. Here we describe the effects of the probiotic LGG on pneumococcal colonization using an *in vitro* adherence assay.

## Results

### Optimization of the pneumococcal adherence assay

As pneumococcal isolates can vary substantially in growth and adherence properties, we selected five pneumococcal isolates representing four serotypes with different clinical characteristics and origins (Table 1). All five isolates had similar growth kinetics: the mid-log phase was determined to be at five hours post-inoculation and stationary phase was reached between 12 to 15 hours post-inoculation (data not shown). The optimal multiplicity of infection (MOI), defined as the maximum dose of pneumococci that could be added without inducing cytopathic effects, was determined to be ten pneumococci per epithelial cell (data not shown).

**Table 1:** Pneumococcal isolates used in this study

**Isolate**	Serotype*	**Clinical category**	**Origin**
PMP843	19 F	Colonising	USA
PMP558	6B	Colonising	Fiji
PMP812	5	Invasive	Bangladesh
PMP6 (ATCC 6305)	5	ND	ATCC
PMP41	3	Colonising	Fiji

* by the Quellung reaction.

ND: Not defined.

Isolates were tested for their ability to adhere to and invade epithelial cell monolayers. After three hours incubation, adherence ranged from less than 1% (PMP41) to approximately 48% (PMP558) of the inoculum (Table 2). Adherence was variable, particularly for PMP843 at the mid-log phase (234% ± 249%, n=8). Due to the low adherence of PMP41, this isolate was not included in subsequent assays. No significant differences were found between the adherence of mid-log and stationary phase isolates, except for PMP558 (P = 0.016), and so the mid-log phase was selected for use in the adherence assay. Invasion levels were less than 1% for all isolates (Table 2), indicating that the vast majority of cell-associated pneumococci recovered were present on the cell surface.

**Table 2:** Adherence and invasion of pneumococcal isolates

**Isolate**	**Serotype**	**% Adherence**		**% Invasion**	
		**Mid-log**	**Stationary**	**Mid-log**	**Stationary**
PMP843	19 F	234 ± 249	193 ± 14.6	<0.01	<0.01
PMP558	6B	48.1 ± 24.6	3.2 ± 2.1*	0.01 ± 0.009	<0.01
PMP812	5	43.8 ± 19.3	15.0 ± 20.3	0.9 ± 1.6	0.1 ± 0.2
PMP6	5	3.1 ± 1.3	1.1 ± 0.2	0.01 ± 0.007	<0.01
PMP41	3	0.03 ± 0.03	0.6 ± 1.0	<0.01	<0.01

Mean and standard deviation of adherence and invasion of pneumococcal isolates at mid-log and stationary growth phase, expressed as percentage of the inocula. n≥3 for all data except PMP6 in stationary phase (n=2). * Significant difference between mid-log and stationary phase (P = 0.016).

**Figure 1:**
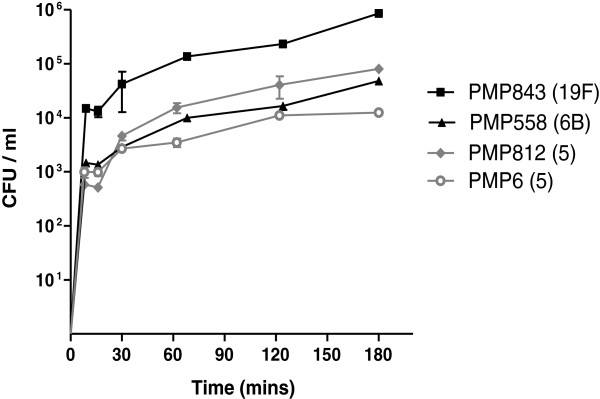
**Time-course of pneumococcal adherence to epithelial cells.** Cells were inoculated with 7.2 x 10^5^ CFU (95%CI 5.8 x 10^5^, 8.7 × 10^5^ CFU) and the number of adherent pneumococci measured over three hours. Mean ± SD for four pneumococcal isolates are depicted (n=2).

An examination of pneumococcal adherence kinetics revealed that adherence occurred rapidly, with more than 10^2^ CFU/ml adherent bacteria detected by eight minutes after inoculation for all isolates examined (Figure 1). The rate of adherence slowed after 60 minutes; at this point adherence had reached a mean of 86.0% (95%CI: 84.9, 87.0) of the maximum for the four isolates, using log transformed data.

### Effect of LGG on pneumococcal adherence

**Figure 2:**
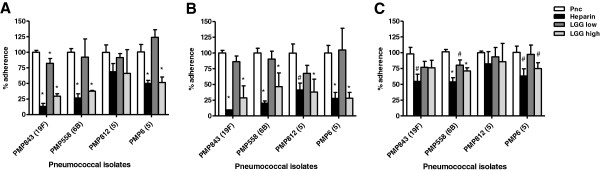
**Effect of LGG on pneumococcal adherence to epithelial cells.** Pneumococcal adherence was determined when incubated with medium alone (Pnc), or with medium containing 100 U/ml heparin (Heparin), or ~5 x 10^6^ CFU LGG (LGG low), or ~5 x 10^7^ CFU LGG (LGG high) added one hour before (**A**), concurrently (**B**), or one hour after adding pneumococci (**C**). One-way ANOVA revealed significant differences in adherence levels (P < 0.05) for all isolates except PMP812 (serotype 5) in the post-addition assay. For each isolate, Bonferroni’s post-test was used to compare heparin, LGG low, and LGG high to Pnc: *, P < 0.001; #, P < 0.05. Data are mean + SD (n≥3).

To investigate the effect of LGG on pneumococcal adherence, we tested a high dose (4.8 x 10^7^ CFU; 95%CI: 3.9 x 10^7^, 5.8 x 10^7^) and low dose (4.8 x 10^6^ CFU; 95%CI: 3.9 x 10^6^, 5.8 x 10^6^ CFU) of LGG, corresponding to a 66:1 and 6.6:1 ratio of LGG: pneumococci, respectively. LGG was added to the cells at one hour before (pre-addition), with (co-addition), or one hour after (post-addition) the pneumococci. Heparin, which blocks pneumococcal adherence to cell surface glycosylaminoglycans [19], was used as a positive control. The high dose of LGG significantly inhibited adherence of three pneumococcal isolates (PMP843, PMP558, and PMP6) in the pre-addition assay (Figure 2A), all four isolates in the co-addition assay (Figure 2B), and two isolates (PMP558 and PMP6) in the post-addition assay (Figure 2C). The inhibitory effect of the higher dose of LGG was greater when administered before or at the same time as pneumococci: when data for all four isolates were pooled, % adherence was 46.5 ± 24.1 for the pre-addition assay, 35.4 ± 17.5 for co-addition, and 77.0 ± 15.2 for post-addition (P<0.0001). The low dose of LGG only significantly inhibited adherence of PMP843 in the pre-addition assay (Figure 2A) and PMP558 in the post-addition assay (Figure 2C).

### Mechanistic studies

To examine the mechanism(s) by which LGG inhibits pneumococcal adherence, we tested whether LGG adheres to epithelial cells and may potentially compete for binding. Following a three hour incubation with a 3.8 x 10^7^(95%CI: 1.5 x 10^7^, 6.1 x 10^7^) inoculum, 11.7 ± 1.4% of LGG adhered to the cells. The adherence of LGG was not affected by the presence of heparin (12.9 ± 0.6%; P>0.05). Co-culturing pneumococci for three hours with high or low doses of LGG in the absence of epithelial cells had no effect on pneumococcal growth (data not shown). To determine if soluble compounds present in the assay media could affect pneumococcal adherence, assay media was collected from epithelial cells incubated with LGG alone or LGG with pneumococci for three hours and filtered to remove bacteria, cells and debris. These cell-free supernatants did not significantly inhibit pneumococcal adherence in subsequent assays (Table 3).

**Table 3:** Effect of culture supernatants on pneumococcal adherence

**Isolate**	**Serotype**	Supernatants*	
		**LGG**	**LGG + Pnc**
PMP843	19 F	103.4 ± 10.3%	85.8 ± 1.6%
PMP558	6B	79.4 ± 26.5%	95.5 ± 12.0%
PMP812	5	127.6 ± 40.7%	140.1 ± 25.6%
PMP6	5	69.0 ± 43.0%	178.1 ± 26.9%

* Culture supernatants were obtained from wells in the cell adhesion assay that contained epithelial cells incubated with LGG alone (LGG), or together with pneumococci isolate (LGG + Pnc). Supernatants were filtered and added to wells in a separate adherence assay to measure their effect on the adherence of the corresponding pneumococcal isolate. Data were normalized to control (pneumococcal adherence when no supernatants added). n=2, P>0.05.

**Figure 3:**
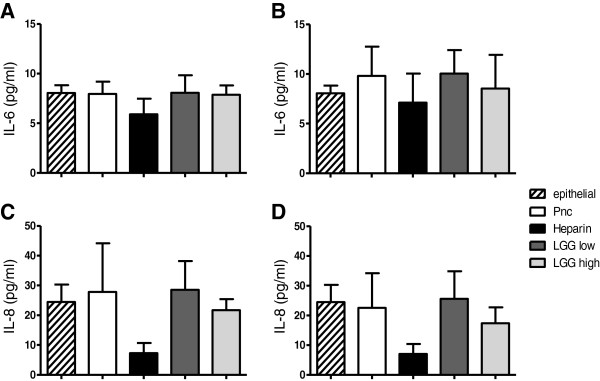
**Effect of LGG on epithelial cytokine production.** Concentrations of IL-6 and IL-8 in culture supernatants of untreated epithelial cells (epithelial) or epithelial cells incubated with pneumococci (Pnc), pneumococci with 100 U/ml heparin (Heparin); or~5 × 10^6^ CFU LGG (LGG low), or ~5 × 10^7^ CFU LGG (LGG high) added one hour prior to the addition of pneumococcal isolates PMP843 (serotype 19 F) (**A** and **C**) and PMP6 (serotype 5) (**B** and **D**) in the adherence assay. Data reported as mean + SD (n=3).

To investigate if LGG could inhibit pneumococcal adherence by modulating the host cytokine response, we measured a panel of cytokines and chemokines (IL-1β, TNF-α, IL-6, and IL-8) in the cell-culture supernatants of the pre-addition adherence assay of PMP6 and PMP843. Only IL-6 and IL-8 were present in detectable levels, and neither was affected by the presence of LGG (Figure 3).

## Discussion

To assess whether the probiotic LGG could prevent colonization of *S. pneumoniae in vitro*, we tested its effect on the adherence of four pneumococcal isolates using an *in vitro* adherence assay. A fifth isolate, PMP41 (a serotype 3 strain), was excluded from the study due to low adherence. This was not unexpected given that serotype 3 is heavily encapsulated, which can result in low adherence [20]. Results demonstrated that LGG inhibits pneumococcal adherence *in vitro*. Inhibition was most effective when LGG was added at a dose approximately 60-fold higher than pneumococci before pneumococcal adherence has been established.

Mechanistic studies demonstrated that LGG binds to epithelial cells, suggesting that LGG could prevent pneumococcal adherence either through steric hindrance or competition for binding sites. Heparin did not affect LGG adherence to epithelial cells, indicating that LGG does not compete for binding to glycosylaminoglycans. However, LGG could compete for binding to other molecules such as fibronectin and collagen, as both *S. pneumoniae* and *Lactobacillus* species have been shown to bind to these molecules [21-24]. Some probiotics are known to produce secreted compounds with antibacterial activity on other species [25,26]. Co-culture experiments indicated that LGG does not have any direct effect on pneumococcal growth or viability, nor did secreted products present in culture media impact pneumococcal growth or adherence.

Several studies have reported increased secretion of inflammatory mediators IL-6 and IL-8 following exposure to pneumococci, both *in vitro* and *in vivo*[27-30]. LGG can modulate host cell production of IL-6 [31,32] and IL-8 [33,34] following exposure to pathogens or microbial antigens, such as flagellin, both *in vivo* and *in vitro*. Therefore, we hypothesized that exposure to LGG would reduce IL-6 and IL-8 production by epithelial cells. However, *S. pneumoniae* did not increase IL-8 or IL-6 secretion by epithelial cells, and other inflammatory cytokines and chemokines were undetectable. These data suggest that the inhibition of *S. pneumoniae* adherence to epithelial cells in this study was not due to LGG modulation of IL-6 or IL-8 production by epithelial cells. The lack of effect on cytokine secretion could be due to the relatively short incubation time or lack of co-stimulation. Marriott et al. [35] recently demonstrated that co-culture of pneumococci-primed macrophages, or supernatants from these cultures, with A549 epithelial cells significantly elevated IL-8 secretion, but not when pneumococcal bacteria were added to A549 cells alone, suggesting a critical role for epithelial-macrophage interactions in this response. Although not statistically significant, the reduction in IL-8 levels in the heparin-treated samples observed in our experiments (Figure 3C and 3D) is likely due to binding of IL-8 by heparin [36,37].

Clinical studies investigating the impact of LGG administration on the incidence of otitis media and respiratory tract infections have reported variable outcomes. Rautava et al. found that infants who were given formula supplemented with LGG and *Bifidobacterium lactis* had a lower relative risk of otitis media and recurrent respiratory infections compared to infants given placebo [38]. Administration of an LGG-containing probiotic mixture to women during the last month of pregnancy and to infants (who were given the probiotic mixture in combination with galactooligosaccharides) for six months after birth was associated with a reduction in infant respiratory infections, although no difference in otitis media was observed [39]. Hosjak et al. found that LGG reduced the risk of respiratory and gastrointestinal infections in hospitalized children [40], whereas in children attending day care, LGG treatment reduced the relative risk of upper respiratory tract infections but did not affect lower respiratory tract or gastrointestinal tract infections [41]. Few clinical data on the effects of probiotic treatment on nasopharyngeal carriage of respiratory pathogens are available. Oral administration of an LGG-containing probiotic mixture reduced nasal colonization of Gram-positive pathogens in adults [18] but did not affect nasopharyngeal carriage of *S. pneumoniae* in otitis-prone children [42]. Furthermore, nasal delivery of *Lactobacillus rhamnosus* (strain LB2) did not affect the nasopharyngeal carriage of *S. pneumoniae* in children with secretory otitis media [43]. LGG may provide a more systemic benefit, as lactobacilli have been shown to possess immunomodulatory and vaccine adjuvant properties [44,45]. However, a probiotic known to colonize the respiratory tract, such as *Streptococcus salivarius*[46], may be more likely to prevent colonization of respiratory pathogens.

## Conclusions

The principal finding from our study is that the probiotic LGG can reduce adherence of pneumococci to epithelial cells in an *in vitro* model. As LGG was effective in inhibiting adherence in the pre- and co- addition assays but less so in the post-addition assay, our data suggest that it would be more effective as a preventative strategy. Our findings support the notion that probiotics can be used as an additional strategy to prevent pneumococcal colonization and hence disease in early life. However, more research is needed to increase understanding of the mechanisms of probiotic action and identify what strategies (type of probiotic, mode, dose, and timing of administration) may be most effective in clinical settings.

## Methods

### Bacterial strains, cells and culture conditions

The pneumococcal isolates used in this study are described in Table 1. Bacterial isolates were obtained from our own culture collection or provided with permission from investigators from previous ethically-approved research. As no new sample collection or animal experiments were performed as part of this study, no additional ethics approval was required. *Lactobacillus rhamnosu*s strain GG (LGG) was obtained from Dicoflor capsules (Dicofarm, Italy). Pneumococci and LGG were cultured on horse blood agar (HBA; Oxoid, Australia) and de Man, Rogosa and Sharpe (MRS) agar (Oxoid, England) supplemented with 0.5% L-cysteine, respectively. For the adherence assay, pneumococci and LGG were cultured in Todd-Hewitt broth (Oxoid, England) supplemented with 0.5% yeast extract (Oxoid, England) or MRS broth (Oxoid, England), respectively. The human epithelial cell line CCL-23 was utilized (American Type Culture Collection, USA). Cells were maintained in modified Eagle’s medium (MEM) (Thermo Scientific, USA) supplemented with 10% fetal bovine serum (Thermo Scientific, Australia), 2 mM L-glutamine and 20 mM 4-(2-Hydroxyethyl)piperazine-1-ethanesulfonic acid. The concentration of fetal bovine serum was reduced to 5% for the adherence assays. Bacteria and epithelial cells were grown at 37°C and in 5% CO_2_ for maintenance and all assays.

### Adherence assay

Epithelial cells were seeded overnight at 1.5 x 10^5^ cells /ml in a 24-well tray (Nunc, Denmark). The cells were then washed with prewarmed PBS and 500 μl MEM added. *S. pneumoniae* isolates were grown to mid-log phase, centrifuged at 1820 x *g*, and resuspended in 0.85% NaCl to a concentration of approximately 1 x 10^8^ CFU/ml. A 10 μl inoculum containing 7.2 x 10^5^ CFU (95%CI 5.8 x 10^5^, 8.7 x 10^5^ CFU) was added to the epithelial cells. PBS was used as a negative control and 100 U/ml heparin (Pfizer, Australia) was used as a positive control for blocking pneumococcal adherence. All assay conditions were performed in duplicate wells. The tray was centrifuged at 114 x *g* for five minutes and then incubated for three hours at 37°C, after which the medium was removed and the cells were gently washed three times with pre-warmed PBS to remove non cell-associated bacteria. Cells were lysed with 0.1% digitonin (Sigma-Aldrich, Australia) and viable counts of pneumococci were obtained by serial dilution and duplicate plating on HBA. Pneumococcal adherence was calculated as the percent of the original inoculum recovered at the end of the assay. LGG adherence was determined in a similar manner except that the samples were plated on MRS agar. To measure pneumococcal invasion, after the three hour incubation period, the culture medium was removed and the cells washed and incubated for another two hours with media containing 5 μg/ml penicillin and 100 μg/ml gentamicin to kill extracellular bacteria. The antibiotic-containing medium was then removed, after which the epithelial cells were lysed with digitonin and the number of CFU/ml determined. To determine the effect of LGG on pneumococcal adherence, LGG was added at 4.8 x 10^7^ CFU (95%CI: 3.9 x 10^7^, 5.8 x 10^7^) or 4.8 x 10^6^ CFU (95%CI: 3.9 x 10^6^, 5.8 x 10^6^ CFU) one hour before (pre-addition), at the same time as (co-addition) or one hour after (post-addition) pneumococci were added. Adherence is reported as percentage of adhering bacteria normalized to the ‘pneumococcal-only’ control (no heparin or LGG). Cell-free culture supernatants were prepared by collecting assay media after the 3 h incubation step, removing debris by centrifugation at 1820 x *g* for 3 min before passing resultant supernatants through a 0.22 μm pore size syringe filter (Millipore).

### Detection of cytokines and chemokines by multiplex bead array

Concentrations of IL-1β, TNF-α, IL-6, and IL-8 were determined in epithelial cell culture supernatants following a three hour pneumococcal adherence assay using a multiplex method. Beadmates consisting of Beadlyte anti-cytokine beads and matched anti-cytokine biotinylated reporters were used according to the manufacturer’s protocol (Millipore, USA). In brief, 25 μl of bead preparation were incubated with 50 μl of standards, controls and samples in a 96 well plate overnight with shaking at 4°C. All culture supernatant samples were assayed undiluted in duplicate. The plate was washed twice and incubated for one hour at room temperature with 50 μl/well of detection antibodies, prior to a 30 minute incubation with 50 μl/well of streptavidin-phycoerythrin reagent. The plate was then washed twice and beads re-suspended in 100 μl/well sheath fluid before reading on a Luminex 200 Bio-analyzer (Luminex Corporation, USA). The lower limit of detection for all cytokines/chemokines was 0.13 pg/ml. Data analysis was performed using the LuminexIS 2.3 software (Luminex Corporation, USA).

### Statistical analyses

Data were analysed using GraphPad Prism version 5.04 for Windows (GraphPad Software, USA). All data are presented as mean ± standard deviation (SD) unless otherwise specified. One-way analysis of variance (ANOVA) with Bonferroni’s post hoc test was used to analyze differences between groups for all data except comparisons of mid-log versus stationary growth in adherence and invasion levels, in which case the Mann–Whitney test was used. All experiments were performed on at least three separate occasions, except where otherwise indicated. P < 0.05 was considered significant for all analyses.

## Competing interests

MT is chairman of the Asia Pacific Immunoglobulins in Immunology Expert Group (APIIEG), which is supported by CSL Ltd and is a member of the Nestle Nutrition Institute Medical Advisory Board, Australia/New Zealand. All other authors declare they have no conflicts of interest.

## Authors’ contributions

SSW, ZQT and EMD performed the experiments and drafted the manuscript. EKM, MLKT and RMRB provided advice on the experimental design and critically revised the manuscript. CS and PVL conceived and designed the study and helped draft the manuscript. All authors have read and approved the final manuscript.
